# Transcription Factors That Control Behavior—Lessons From *C. elegans*

**DOI:** 10.3389/fnins.2021.745376

**Published:** 2021-09-27

**Authors:** Rasoul Godini, Ava Handley, Roger Pocock

**Affiliations:** Development and Stem Cells Program, Department of Anatomy and Developmental Biology, Monash Biomedicine Discovery Institute, Monash University, Melbourne, VIC, Australia

**Keywords:** transcription factors, *Caenorabditis elegans*, behavior, neuronal specification and differentiation, neuronal circuit development, sex-specific behavior, sensory systems

## Abstract

Behavior encompasses the physical and chemical response to external and internal stimuli. Neurons, each with their own specific molecular identities, act in concert to perceive and relay these stimuli to drive behavior. Generating behavioral responses requires neurons that have the correct morphological, synaptic, and molecular identities. Transcription factors drive the specific gene expression patterns that define these identities, controlling almost every phenomenon in a cell from development to homeostasis. Therefore, transcription factors play an important role in generating and regulating behavior. Here, we describe the transcription factors, the pathways they regulate, and the neurons that drive chemosensation, mechanosensation, thermosensation, osmolarity sensing, complex, and sex-specific behaviors in the animal model *Caenorhabditis elegans*. We also discuss the current limitations in our knowledge, particularly our minimal understanding of how transcription factors contribute to the adaptive behavioral responses that are necessary for organismal survival.

## Introduction

**FIGURE 1:**
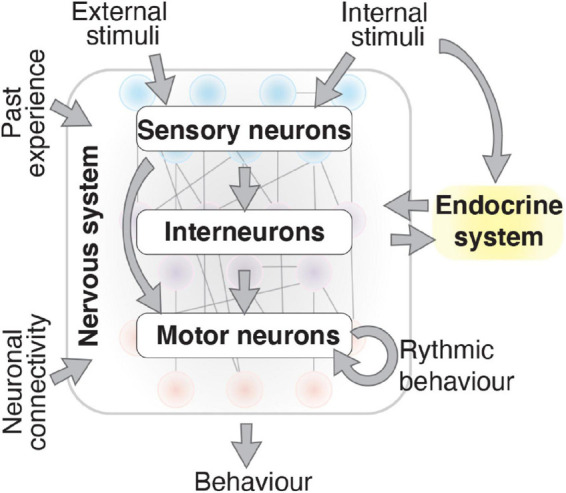
Behavior results by integrating numerous factors. The nervous system produces behavior that is influenced by external and internal stimuli, past experience, neuronal connectivity, and the endocrine system. Information flows from sensory neurons to interneurons and motor neurons through synaptic and gap junction connections and neuropeptide secretion. Consistent rhythmic behaviors, produced by motor neurons, are also influenced by the physiological state of the nervous system caused by internal and external signals.

Organismal survival requires the correct response to internal and external challenges. Behavioral changes are one of the major response mechanisms in animals, and has been defined as the “*Whole living organism’s internally coordinated responses to internal and/or external stimuli, excluding developmental changes*” (Levitis et al., 2009). Behavioral responses are controlled by the nervous and endocrine systems (Gámez-del-Estal et al., 2014; Marlin et al., 2015). In the nervous system, neurons control behavior by integrating and responding to molecular cues, past experience and neuronal connectivity (Figure 1; Bargmann et al., 1993; Ardiel and Rankin, 2010; Yapici et al., 2014; Oren-Suissa et al., 2016). A behavioral response can be adaptive, such as responding to attractive or noxious stimuli via sensory neurons. Behavioral responses may also be rhythmic, such as breathing in vertebrates (Nusbaum and Beenhakker, 2002), or locomotor wave generation in the nematode *Caenorhabditis elegans* (Fouad et al., 2018), which involve intrinsic regulation within motor circuits. Within neurons, sophisticated molecular mechanisms convert stimuli into intracellular signals and enable the stimulus to be transduced throughout the animal (Huang and Chalfie, 1994; Sengupta et al., 1996; Chatzigeorgiou et al., 2010). Studying behavioral responses in multicellular organisms can be challenging, as nervous system complexity and the plethora of intersecting molecular pathways involved make an animal sensitive to subtle environmental or internal changes. A well-characterized nervous system and the ability to tightly control environmental conditions can enable behavioral mechanisms to be experimentally dissected. *C. elegans* possess both advantages, making it a valuable model organism to study behavior.

*C. elegans* has a small nervous system, hermaphrodites and males have 302 and 385 neurons, respectively, with 294 neurons common between the sexes (Hobert, 2005). The function of many *C. elegans* neurons has been identified by ablating specific neurons and examining whether the worm can perform a certain behavior (Bargmann and Avery, 1995; Fang-Yen et al., 2012). In addition to understanding the developmental trajectory, position and function of individual neurons, *C. elegans* is the only animal with a completely mapped connectome—a map of synaptic connections between neurons (White et al., 1986; Jarrell et al., 2012; Cook et al., 2019). The transcriptome of almost all neurons has also been measured at several developmental stages (Cao et al., 2017; Packer et al., 2019; Taylor et al., 2021). These data allow the gene expression profile and the function of specific neurons to be linked, providing an unparalleled opportunity for understanding the molecular mechanisms that control behavior at single cell resolution.

Key regulatory factors that define the transcriptome and identity of neurons are transcription factors (TFs). More than 900 TFs from different families have been predicted in *C. elegans* (Reece-Hoyes et al., 2005; Haerty et al., 2008; Narasimhan et al., 2015). Some of these TFs are highly conserved and have many orthologs in other animals, such as members of the homeodomain, forkhead, and zinc finger families (Narasimhan et al., 2015). TFs play important roles in development (Hobert, 2008; Bertrand et al., 2011), the immune response (Ooi and Prahlad, 2017), aging (Murphy et al., 2003), sex-determination (Hodgkin and Brenner, 1977; Berkseth et al., 2013), and regulating the development and function of neuronal circuits (Sengupta et al., 1996; Kim et al., 2010; Oren-Suissa et al., 2016). By regulating neuron development, neuronal connectivity and sex-specificity, TFs perform a fundamental role in orchestrating behavioral responses. Here, we review the function of TFs in *C. elegans* behavior. Recent genomics data reveal that many hundreds of TFs are expressed in neurons (Cao et al., 2017; Packer et al., 2019; Taylor et al., 2021). However, most of these have yet to be studied in relation to neuron function and behavior and so will not be discussed here. In this review, we focus on those TFs that have been functionally shown to play a role in behavior, paying particular attention to those TFs that do not disrupt overall neuronal morphology, but play a more defined role in regulating behavior. We describe how TFs regulate fate determination and control molecular mechanisms in different neuron types involved in behavior and discuss how TFs regulate sex-specific behavior.

### Transcription Factors Driving Neuron Identity

Behavior requires the coordinated output of different cell types that each possess a distinct structure, location, connectivity, and molecular identity. TFs define cell identity by regulating the gene expression program within that cell. Some features of neuronal identity are pan-neuronal, such as synaptic vesicle loading and neuropeptide secretion. These pan-neuronal identities are controlled by redundant regulatory inputs, including multiple TFs such as from the homeodomain family, that coordinate gene regulation through multiple regulatory elements (Stefanakis et al., 2015). Neuron-specific identities, those features that provide neurons with their individual characteristics, are defined by “terminal selector” TFs (Hobert, 2008). Terminal selectors for more than 70% of extra-pharyngeal neurons have been identified (for a comprehensive review see Hobert, 2016), and they regulate neuron identity either alone or in combination with other TFs. For example, a complex comprising TTX-3 [LIM homeobox 9 (LHX9) ortholog], and CEH-10 [human visual system homeobox 2 (VSX2) ortholog], activates another *C. elegans* homeobox TF CEH-23, to control the identity of the AIY interneurons (Altun-Gultekin et al., 2001).

Terminal selectors can be very specific to individual neuron types or can cooperate to determine the fate of a group of neurons. For example, the nuclear-hormone receptor type TF ODR-7 acts specifically in the AWA sensory neurons (Sengupta et al., 1994), whereas the E-twenty-six (Ets) domain TF AST-1 controls the expression of all dopamine pathway genes in dopaminergic neurons (Flames and Hobert, 2009). Cooperating with AST-1 is the distal-less homeobox TF CEH-43, which is required and sufficient for dopaminergic neuron development. Additional TFs can define a more specific identity within a neuronal subgroup, for example CEH-20, a PBX TF, is required for differentiating the PDE neuron, a dopaminergic neuron in the midbody (Flames and Hobert, 2009; Doitsidou et al., 2013). Particular glutamatergic neurons require specific terminal selectors for their identity, such as CHE-1, a zinc finger TF in the ASE neurons (Uchida et al., 2003), and ETS-5 in the BAG neurons (Guillermin et al., 2011; Brandt et al., 2012). In contrast, the TFs UNC-86, LIN-11, and CEH-14 are expressed in multiple glutamatergic neurons but are terminal selectors for only some of them (Sarafi-Reinach et al., 2001; Serrano-Saiz et al., 2013). For example, CEH-14 is necessary for PHA, PHB, and PHC glutamatergic identity, but is also expressed in PVQ and PVR glutamatergic neurons (Serrano-Saiz et al., 2013). The TFs involved in defining cholinergic and serotonergic identities, TTX-3, UNC-3, and UNC-86, can also act either individually or in combination, depending on the neuron (Prasad et al., 2008; Kratsios et al., 2012; Zhang et al., 2014). For example, TTX-3 alone controls AIA cholinergic interneuron fate, but both TTX-3 and UNC-86 cooperate to drive serotonergic NSM identity (Zhang et al., 2014). UNC-86 also cooperates with the CFI-1, an AT-rich interaction domain (ARID) TF, to control IL2 and URA cholinergic identity (Zhang et al., 2014). In *C. elegans*, some neurons may possess dual neurotransmitter identity, in that the gene expression programs that produce particular neurotransmitters are activated in the same neuron. For example, the AIM interneuron and ASG sensory neurons can be glutamatergic, driven by LIN-11, as well as serotonergic, driven by HIF-1 (Pocock and Hobert, 2010; Serrano-Saiz et al., 2013). The small set of TFs described so far are involved in establishing neuronal identity. In the following sections, we will expand on the TF repertoire that is necessary for establishing the specific behavioral function of neurons.

### Transcription Factors Driving Sensory Mechanisms

Sensory neurons are the first responders to environmental signals. Sensory systems are categorized into several groups depending on the stimulus: chemical, mechanical, osmotic, and thermal (Cassata et al., 2000; Yu et al., 2017). Each one of these sensory systems comprises specific sensory neurons that express molecules, controlled by TFs, that allow them to sense specific stimuli.

#### Chemosensation

*C. elegans* possess a powerful chemosensory system for perceiving chemicals in the environment, including food, noxious elements, volatile compounds, gases, and mating signals (Bargmann et al., 1993; Troemel et al., 1997; Chang et al., 2006; White et al., 2007; Ryan et al., 2014). Chemosensation is important for a wide range of *C. elegans* behavior including chemotaxis, avoidance, and motility. Chemosensory neurons control behavior by signaling to downstream inter- or motor- neurons, and other tissues. In addition to controlling behavior, chemosensory neurons can regulate animal physiology and development by releasing secreted TGF-β-family neuropeptides. For example, the ASI chemosensory neurons can secrete DAF-7, a TGF-beta related peptide, to control dauer entry (Ren et al., 1996; Schackwitz et al., 1996).

##### Chemical (Odorant) Sensing

**FIGURE 2:**
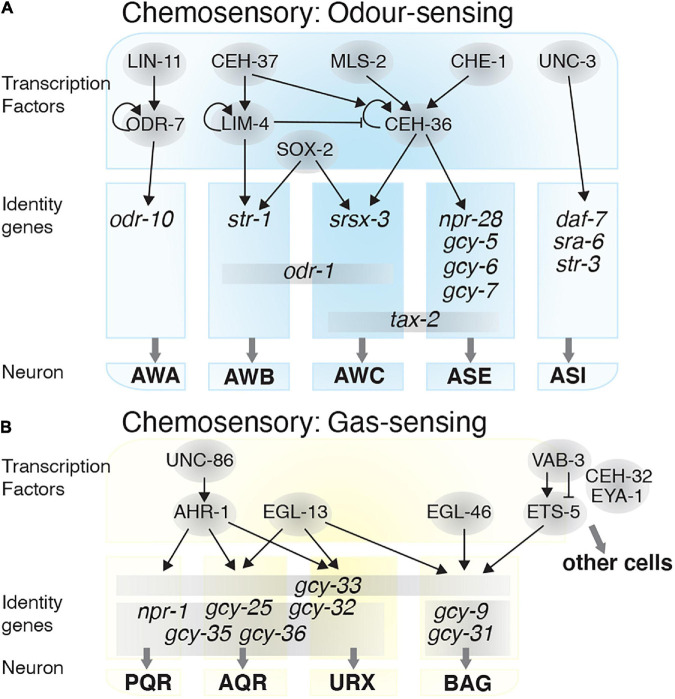
Transcriptional regulatory networks in chemosensory neurons. Transcription factors are depicted in gray circles. How these transcription factors interact and the identity genes they regulate are shown for the: **(A)** Odor-sensing neurons and **(B)** gas-sensing neurons.

The AWA, AWB, and AWC olfactory neurons sense volatile attractants from food and chemicals. Depending on the characteristics of the compound being sensed, these neurons can mediate attractive or repulsive behavior (Bargmann et al., 1993; Troemel et al., 1997). Correct diversification of these neurons is vital for the olfactory system to function efficiently. Several TFs act, either individually or in combination, to induce or repress specific neuron identities. AWC identity requires three TFs: CEH-36, MLS-2, and SOX-2. MLS-2 initiates *ceh-36* expression during post-mitotic development (Kim et al., 2010; Alqadah et al., 2015). CEH-36 and SOX-2 then cooperate to drive expression of *odr-1* (receptor-type guanylate cyclase), *srsx-3* (G protein-coupled receptor domain), and *tax-2* (cyclic nucleotide-binding domain protein), genes required for AWC chemosensory identity (Figure 2A; Kim et al., 2010; Alqadah et al., 2015). The T-box family TF, TBX-2, is also required for olfactory adaptation by the AWC neurons but has no overt role in AWC development or differentiation (Miyahara et al., 2004).

AWA chemosensory neuron differentiation occurs when *odr-7* represses AWC fate. During early AWA development, LIN-11 induces *odr-7* expression, which thereafter autoregulates (Sarafi-Reinach et al., 2001). ODR-7 regulates *odr-10*, an odorant receptor, which is required for AWA-mediated chemotaxis (Sengupta et al., 1994, 1996). AWB-specific identity is established through the TF LIM-4, and both AWB and AWC require SOX-2 for their fate determination. In the AWB neurons, cooperation between SOX-2 and LIM-4 induces AWB fate by expressing the *str-1* and *odr-1* receptors, and repressing AWC fate. Without LIM-4 the AWBs, which normally mediate repulsive behavior, switch to an AWC-like attractive behavior (Sagasti et al., 1999; Alqadah et al., 2015). LIM-4 expression is itself regulated by the homeobox TF CEH-37, which is required for AWB fate determination and avoidance responses to the volatile repellent 2-non-anone (Troemel et al., 1997). However, AWB neurons lacking CEH-37 retain generic sensory properties and do not convert to an alternate fate (Figure 2A; Lanjuin et al., 2003).

Other well-studied chemosensory neurons are the ASE and the ASI neurons. The ASE neurons mediate attraction toward water soluble chemicals such as Cl^–^, Na^+^, cAMP, and biotin (Bargmann and Horvitz, 1991). The zinc finger TF, CHE-1 drives the fate and chemotaxis properties of the ASE neurons by inducing expression of receptor proteins including NPR-28, GCY-5, GCY-6, GCY-7, and the TAX-2 cation channel (Chang et al., 2003; Uchida et al., 2003; Etchberger et al., 2009). CEH-36, which controls AWC fate, is also required for establishing ASE neuron chemosensory function. CHE-1 controls *ceh-36* expression, and loss of *ceh-36* leads to reduced *gcy-7* and *tax-2* expression in the ASE neurons (Figure 2A; Koga and Ohshima, 2004).

The ASI neurons are involved in chemotaxis and pheromone sensing (Bargmann and Horvitz, 1991; White and Jorgensen, 2012). ASI neuron fate and functionality is determined by UNC-3, a member of the Collier/Olf1/EBF (COE) TF family. UNC-3 is required for expression of the receptors SRA-6 and STR-3, and the TGF-beta peptide DAF-7, while repressing other fate programs in the ASI neurons. For example, *unc-3* mutants show aberrant *odr-10*, *ceh-36*, *flp-20*, and *gcy-7* expression in the ASI neurons (Figure 2A; Kim et al., 2005), and present dauer-regulatory defects due to mis-expression of *daf-7* (Prasad et al., 1998).

##### Gas Sensing

In addition to odorants, *C. elegans* also use chemosensory neurons to detect changes in O_2_ and CO_2_ levels. Gas sensing neurons, such as BAG, URX, AQR, and PQR (Chang et al., 2006; Zimmer et al., 2009), enable the worm to avoid low or high O_2_ levels, thereby protecting the animal from hypoxia or hyperoxia (Figure 2B). Heme-binding proteins, named guanylate cyclases, mediate O_2_/CO_2_ sensing. Worms use guanylate cyclases such as GCY-31, GCY-33, GCY-35, and GCY-36 (Zimmer et al., 2009), to sense changes in O_2_ concentration, and alter their motility and social feeding behavior accordingly (Gray et al., 2004). The AQR, PQR and URX neurons mediate social feeding behavior through the neuropeptide receptor NPR-1 (De Bono and Bargmann, 1998). NPR-1 expression is controlled by the TF AHR-1, which itself is regulated by UNC-86, and loss of *ahr-1* function leads to defects in social feeding behavior (Qin and Powell-Coffman, 2004). The TF EGL-13 is also required for BAG, URX, AQR, and PQR neuron fate determination, and as such *egl-13* mutants are defective for O_2_/CO_2_ sensing (Petersen et al., 2013). The zinc-finger TF EGL-46 and ETS-domain TF ETS-5, are involved in determining the BAG neurons ability to sense O_2_ and CO_2_ (Guillermin et al., 2011; Brandt et al., 2012; Romanos et al., 2015). ETS-5 expression is controlled by VAB-3, of which there are several isoforms. One isoform contains a paired domain and a homeobox domain, which represses *ets-5* expression in other cells, and one isoform containing only the homeobox domain enhances *ets-5* expression in the BAG neurons (Brandt et al., 2019). Two other TFs, CEH-32 and EYA-1, also indirectly associate with VAB-3 to repress *ets-5* (Figure 2B; Brandt et al., 2019).

#### Mechanosensation

Mechanosensory neurons sense external forces and internal tension generated by movement and convert them into electrical signals, through mechanotransduction (Goodman, 2006; Goldmann, 2014). Mechanotransduction is mediated by several mechano-electrical transduction ion channels, including proteins from the TRP and Degenerin/epithelial Na^+^ (DEG/ENaC) channel families. MEC-4 and MEC-10 are members of the (DEG/ENaC) channel family that are required for responding to gentle touch (Chalfie and Sulston, 1981; Huang and Chalfie, 1994; O’Hagan et al., 2005; Chatzigeorgiou et al., 2010). In *C. elegans*, several mechanosensory neurons are responsible for sensing touch, including the anterior AVM and ALMs, and posterior PVM and PLMs (Chalfie et al., 1985). Distinct neurons are involved, and consequently different behaviors are executed, depending on the severity or the location of the mechanical stimulus.

**FIGURE 3:**
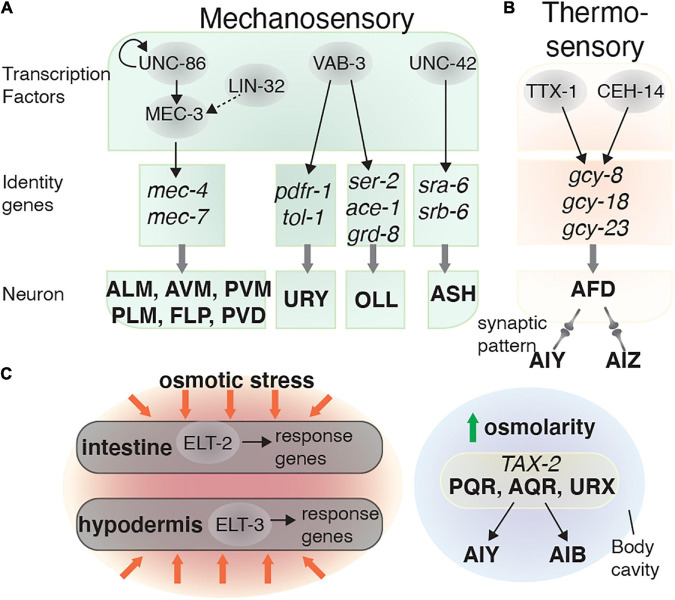
Transcriptional regulatory networks in mechanosensory and thermosensory neurons. Transcription factors are depicted in gray circles. How these transcription factors interact and the identity genes they regulate are shown for the: **(A)** Mechanosensory and **(B)** AFD (thermosensory) neurons. **(C)** Osmolarity sensation is mediated by neurons and non-neuronal tissues (intestine and hypodermis).

Several TFs control terminal differentiation of mechanosensory neurons and touch response behaviors. MEC-3, a LIM-homeobox TF, is expressed in several mechanosensory neurons, including AVM, ALMs, PVM, PLMs, FLPs, and PVDs and is required for touch response behavior (Way and Chalfie, 1989). The UNC-86 and LIN-32 TFs are the major developmental regulators of *mec-3*-expressing neurons. Both of these factors are required to develop the precursor lineage of sensory touch neurons, and likely regulate *mec-3* expression indirectly (Way and Chalfie, 1989). MEC-3 and UNC-86 form a heterodimer that regulates *mec-3* expression and two other genes required for mechanotransduction, *mec-4* and *mec-7* (Figure 3A; Way and Chalfie, 1989; Duggan et al., 1998).

The ASH sensory neurons are involved in several behaviors, including light touch response and odor avoidance (Kaplan and Horvitz, 1993; Troemel et al., 1995). The Prop1-like homeobox domain TF UNC-42 is required for mechanosensation and locomotion by regulating the terminal differentiation of several neurons, including the ASH neurons (Baran et al., 1999). While UNC-42 is not essential for determining ASH sensory identity, it partially controls the terminal differentiation of ASH by regulating expression of the putative chemosensory receptors SRA-6 and SRB-6 (Figure 3A). UNC-42 is also expressed in the AVA and AVD command interneurons, which are important for controlling rapid locomotion and the body touch response (Brockie et al., 2001; Wightman et al., 2005). When UNC-42 function is abolished, this leads to body touch defects (White et al., 1986; Baran et al., 1999).

Several other TFs can also control mechanosensory neuron development. LIN-32, EGL-5, and VAB-15 are involved in generating touch sensory neurons such as the PLM and AVM/PVM, and are therefore required for touch sensitivity (Chalfie and Au, 1989; Du and Chalfie, 2001). Other transcriptional regulators such as EGL-44, EGL-46, and SEM-4 repress touch receptor identity. For example, removing either *egl-44* or *egl-46* function results in neurons emerging with touch receptor identity that are absent in the wild-type animals (Mitani et al., 1993). In addition to controlling *ets-5* expression in the BAG neurons, VAB-3 also controls other glutamatergic sensory neuron identities. This includes the potential mechanosensory neurons OLL (Perkins et al., 1986), where VAB-3 regulates *eat-4*, *ser-2*, *ace-1*, and *grd-8* expression (Serrano-Saiz et al., 2013). However, specific behavioral defects in *vab-3* mutants have not been reported, possibly due to their severe morphological defects (Figure 3A).

### Thermosensation and Osmotic Responses

*C. elegans* can sense and adapt to environmental temperature changes through processes known as thermosensation and thermotaxis memory behavior. Thermosensation is mediated by the AFD sensory neurons, and the AIY and AIZ interneurons (Cassata et al., 2000). The AFDs sense temperature using a group of guanylate cyclases: GCY-8, GCY-18, and GCY-23 (Inada et al., 2006). The TFs CEH-14 and TTX-1 are required for the final step of AFD differentiation, by inducing *gcy-8* and *gcy-18* expression (Cassata et al., 2000; Kagoshima and Kohara, 2015). The cooperation of these two TFs is important for AFD fate determination, as ectopically expressing both *ceh-14* and *ttx-3* in the AWB neurons induces an AFD-fate, whereas expressing them individually does not (Figure 3B; Kagoshima and Kohara, 2015).

Regulating intracellular osmolarity is critical for maintaining homeostasis. In *C. elegans*, osmoregulation is mediated by the intestine, hypodermis, and excretory cell (Nelson and Riddle, 1984; Rohlfing et al., 2010). The GATA family TFs ELT-2 and ELT-3 are required for controlling osmotic stress responses in the intestine and hypodermis, respectively (Figure 3C; Rohlfing et al., 2010). In the nervous system, the cGMP-gated channel subunit TAX-2, expressed in the AQR, PQR, and URX neurons, mediates the response to mild upshifts in osmolarity (Yu et al., 2017). The AQR, PQR, and URX neurons can directly sense osmotic alterations in body fluid within the body cavity (White et al., 1986), and these neurons then send signals to the AIB and AIY interneurons, which in turn control animal locomotion (Figure 3C; Yu et al., 2017). Knowledge is very limited about the TFs involved in defining the neural circuits involved in osmotic responses and requires further study.

### Transcription Factors That Control Information Processing and Locomotion

After environmental signals are perceived by sensory neurons, this information must be processed and transferred to downstream neurons or other cells to induce the appropriate behavioral response. Interneurons are the main connection between sensory information and behavioral response. Depending on their circuitry, interneurons are involved in distinct behaviors, including complex behavior and learning which we describe in a separate section below. Motor neurons form the last layer of neuronal circuitry, inducing the locomotion associated with the behavior. Here we describe the TFs that establish inter- and motor-neuron identity and connectivity.

#### Interneurons

**FIGURE 4:**
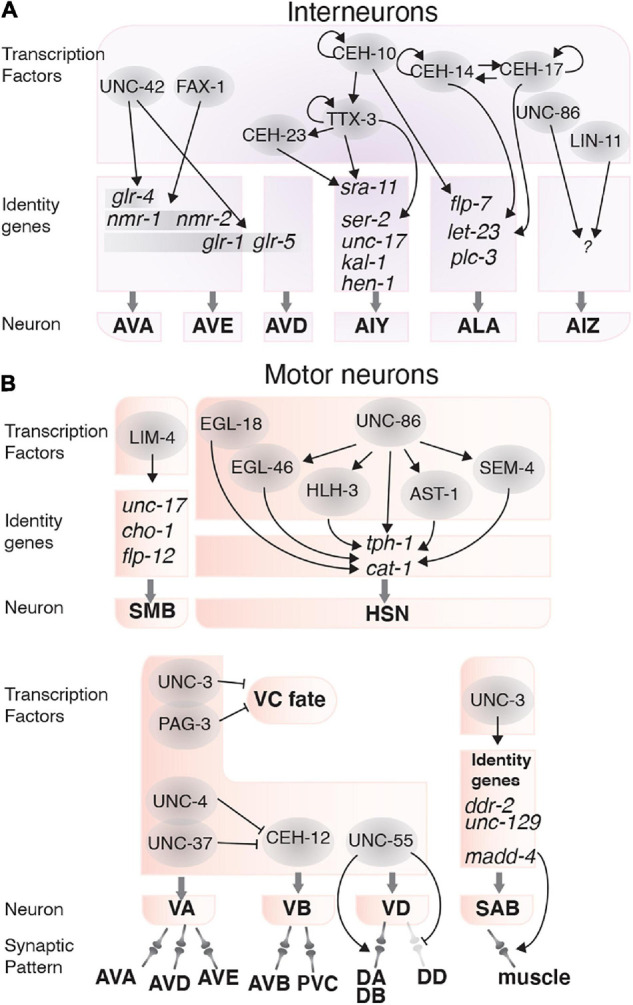
Transcription factors involved in interneuron and motor neuron function. **(A)** Transcription factors (gray circles) and their targets that determine interneuron fate and function. **(B)** Transcription factors that control locomotion by regulating the fate, function and connectivity of motor neurons.

The AVA, AVD, and AVE are command interneurons that mediate backward locomotion (White et al., 1986; Schafer, 2015). The AVA and AVE interneuron identities are regulated by FAX-1 and UNC-42 in complementary and overlapping pathways. FAX-1, a nuclear hormone receptor, induces expression of the NMDA-type glutamate receptor subunits NMR-1 and NMR-2 (Wightman et al., 2005). UNC-42 controls expression of the AMPA-type glutamate receptor subunits *glr-1*, *glr-5*, and *glr-4*. UNC-42 regulates *glr-1* expression in the AVA, AVE, and AVD interneurons to enable the nose touch response (Baran et al., 1999; Brockie et al., 2001). UNC-42 also induces *glr-5* expression in the AVA, AVE, and AVD interneurons, and induces *glr-4* expression solely in the AVA interneurons (Baran et al., 1999; Brockie et al., 2001; Wightman et al., 2005). UNC-42, but not FAX-1, also regulates axon guidance in the AVA, AVD, and AVE neurons (Figure 4A; Brockie et al., 2001; Wightman et al., 2005). Therefore, UNC-42 and FAX-1 control different pathways that define how the AVA, AVD, and AVE command interneurons can respond to different contextual inputs from upstream neurons.

The AIY interneurons receive and integrate information from several amphid sensory neurons, including AWA, AWB, AFD, and ASE, and are involved in locomotion, thermotaxis and chemotaxis (Mori and Ohshima, 1995; Tsalik and Hobert, 2003; Gray et al., 2005; Kuhara et al., 2011; Kocabas et al., 2012). The AIY and AIZ interneurons mediate thermosensory behavior by connecting the AFD thermosensory neurons to command motor neurons. Three homeodomain TFs, TTX-3, CEH-10, and CEH-23 control AIY neuron terminal differentiation. Among these, TTX-3 is required for thermosensory behaviors (Hobert et al., 1997). These TFs regulate each other linearly, such that CEH-10 regulates *ttx-3* and TTX-3 regulates *ceh-23*. Additionally, TTX-3 and CEH-10 autoregulate their expression (Hobert et al., 1997; Forrester et al., 1998; Altun-Gultekin et al., 2001). TTX-3 also regulates expression of surface receptors and channels including *ser-2*, *kal-1*, *unc-17*, *hen-1*, and the serpentine receptor *sra-11*, which is also induced by CEH-23 (Figure 4A; Altun-Gultekin et al., 2001). Loss of TTX-3 function also causes defects in AIY neuron axon outgrowth and pathfinding (Hobert et al., 1997). Thus, TTX-3 controls thermosensory behaviors by establishing and maintaining AIY interneuron function.

The AIZ interneurons are involved in the odorant sensing pathway and receive signals from the AWA and AWC chemosensory neurons (Bargmann et al., 1993). The POU/homeobox TF UNC-86 controls AIZ development and is required for odor-attraction and odor-adaptation behavior. UNC-86 induces AIZ generation during embryogenesis and is required to maintain AIZ function throughout life. LIN-11 also acts in the AIZ interneurons to regulate odor sensing behavior and is required for AIZ axonal morphology but is not involved in AIZ fate determination (Figure 4A; Hobert et al., 1998; Sze and Ruvkun, 2003).

The ALA interneuron and the RIS GABAergic interneuron regulate a sleep-like behavior known as lethargus quiescence (Van Buskirk and Sternberg, 2007; Turek et al., 2013). Two homeodomain TFs, CEH-14, and CEH-17, collaborate to control ALA fate determination and are therefore required for lethargus quiescence (Van Buskirk and Sternberg, 2010). CEH-14 and CEH-17 induce the expression of *let-23*, a receptor tyrosine-protein kinase and *plc-3* (phospholipase C) in the ALA neuron. LET-23 is activated by the epidermal growth factor-like LIN-3, which initiates a signaling pathway that inhibits pharyngeal pumping and locomotion (Van Buskirk and Sternberg, 2007). CEH-10 is also partially involved in lethargus quiescence, by regulating expression of *plc-3*, and the neuropeptide *flp-7* (Van Buskirk and Sternberg, 2010). CEH-10, CEH-14, and CEH-17 also regulate ALA axon outgrowth, which is dispensable for lethargus quiescence (Van Buskirk and Sternberg, 2007). Finally, the RIS interneuron requires the APTF-1 TF, which induces locomotion quiescence through neuropeptide signaling (Figure 4A; Turek et al., 2013).

#### Motor Neurons

Motor neurons generate behavior-specific movements through neurotransmitter and neuropeptide release. Motor neuron outputs can be sex-specific, such that males generate mating-specific movements (Liu et al., 2007; Sherlekar et al., 2013; Mowrey et al., 2014; Choi et al., 2015), and can control rhythmic behaviors, such as egg-laying via HSN (hermaphrodite-specific neuron)-regulated vulval muscle contraction in hermaphrodites (Waggoner et al., 1998). In addition to correct fate determination, synaptic patterns are also essential for correct motor neuron diversity and function (Zhou and Walthall, 1998). Several TFs (detailed below) are critical for locomotion and are required for motor neuron fate determination or synapse formation.

TFs involved in motor neuron fate determination include LIM-4, an LHX6 ortholog, which determines SMB motor neuron fate. LIM-4 regulates *flp-12*, *unc-17*, and *cho-1* expression, which are required for SMB function (Kim et al., 2015). The TF UNC-3 controls fate determination and synaptogenesis of the SAB motor neurons, which potentially controls head and neck movements in L1 larvae (Kerk et al., 2017). UNC-3 regulates SAB fate by activating *ddr-2* and *unc-129* expression, and mediates synaptogenesis by controlling *madd-4*, a secreted protein that organizes synapse formation by controlling AChR clustering on the muscle (Kratsios et al., 2015). Lack of *unc-3* leads to defects in synapse formation between the SABs and head muscles (Kratsios et al., 2015). The VA and the VB motor neurons control backward and forward movements, respectively (Chalfie et al., 1985). The TFs UNC-3 and PAG-3 are expressed in the VA and VB motor neurons and are required for coordinated movements in *C. elegans* (Jia et al., 1996; Cameron et al., 2002). UNC-3 and PAG-3 collaborate to determine VA and VB neuron fate by suppressing VC fate in these neurons (Figure 4B; Jia et al., 1996; Cameron et al., 2002; Prasad et al., 2008).

Specific behavioral movements are achieved by forming neuron-specific synaptic connections between different motor neurons and command interneurons. Failure to establish these connectivity patterns causes defective locomotion. Synaptic patterns in the VA and VB neurons are controlled by the TFs UNC-4, UNC-37, and CEH-12 (Von Stetina et al., 2007). UNC-4 and UNC-37 are expressed in VA neurons and suppress CEH-12, a homeobox TF that regulates VB specific genes (Miller and Niemeyer, 1995; Pflugrad et al., 1997). CEH-12 is only expressed in the VB motor neurons and is likely required for generating the VB synaptic pattern (Von Stetina et al., 2007). Loss of UNC-4 leads to CEH-12 expression and induces the VB synaptic pattern in the VA neurons (Von Stetina et al., 2007). UNC-4 and UNC-37 are required for normal locomotion; however, CEH-12 is not, suggesting CEH-12 plays a subtler role in regulating VB neuron traits. Ventral and dorsal motor neurons (VD and DD motor neurons) are also involved in locomotion. The distinct synaptic patterns of these neurons are mediated by UNC-55, a nuclear hormone receptor. UNC-55 is required for synapse formation between the VD motor neurons and the DA and DB motor neurons. Loss of *unc-55* also leads to the VDs acquiring a DD synaptic pattern and locomotory defects (Figure 4B; Zhou and Walthall, 1998).

As mentioned above, motor neurons can control rhythmic behaviors. An example of this are the HSNs, which control egg-laying through G protein-coupled receptor-mediated regulation of vulval muscle contraction (Dong et al., 2000; Ringstad and Horvitz, 2008; Collins et al., 2016). When HSN function is lost, egg-laying is defective, and eggs accumulate in the uterus (Trent et al., 1983). TFs from six different families control HSN neuron terminal differentiation and function. These TFs are: UNC-86 (POU domain), HLH-3 (bHLH domain), EGL-18 (GATA factor), AST-1 (Ets domain), SEM-4 and EGL-46 (zinc finger). Together, these TFs induce and maintain HSN-expressed genes, including *tph-1* and *cat-1*, with UNC-86 acting as the master regulator for most of the other TFs (Lloret-Fernández et al., 2018). All of these TFs are expressed in the HSNs throughout life, except for HLH-3 which is not expressed after the L4 stage (Lloret-Fernández et al., 2018). The intricate regulatory mechanisms in the HSN neurons highlight the complexity of neuronal control of behavior (Figure 4B).

### Transcription Factors and Complex Behaviors, Sleep, Feeding, Learning, and Memory

Behaviors are not always a simple response to a stimulant. Some behaviors result from the collaboration of multiple neuronal circuits and tissues which combine memories of past experiences, new stimulants, and environmental conditions. Prime examples of these complex behaviors are long-term locomotion patterns (Bargmann, 2006), learning and memory formation (Ardiel and Rankin, 2010; Prithika et al., 2017; Dahiya et al., 2019), and pathogenic avoidance behaviors (Lee and Mylonakis, 2017; Ooi and Prahlad, 2017). TFs can be involved in these complex behaviors from controlling neuron differentiation to regulating the expression of specific signaling molecules. The roles of TFs in mammalian memory formation have been reviewed by Alberini (2009). Here we describe how TFs control complex behavior and memory in *C. elegans*.

#### Food Seeking Behavior

**FIGURE 5:**
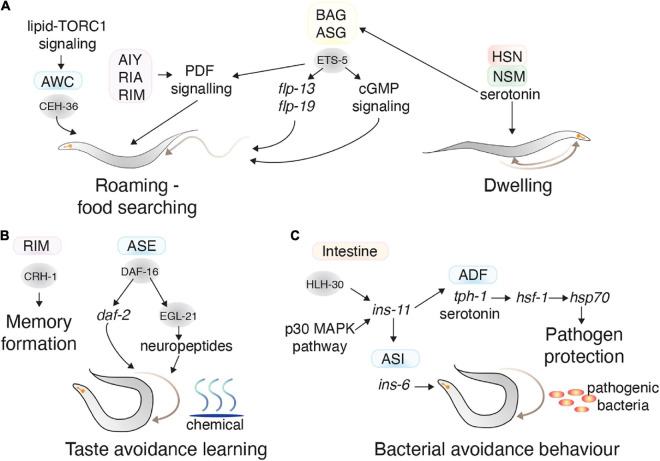
Transcription factors in learning and complex behaviors. **(A)** The TFs (gray circles) and neurons (colored squares) involved in regulating food seeking behavior. CEH-36 in the AWC and ETS-5 in the BAG promote roaming behavior, whereas serotonin secretion from the HSN and NSM promote dwelling behavior. **(B)** The TFs (gray circles) and neurons (colored squares) involved in memory formation and taste avoidance learning. **(C)** The activity of TFs in distal tissues also regulate behavior. HLH-30 and p30 MAPK pathway in the intestine impact bacterial avoidance behavior by regulating *ins-11*. This neuropeptide regulates *ins-6* in the ASI neurons and serotonin in the ADF neurons, which regulate bacterial avoidance behavior.

*C. elegans* locomotion is influenced by feeding and physiological status. *C. elegans* grown on a bacterial lawn typically exhibit three behavioral states: dwelling (feeding in a restricted area), quiescence (a sleep-like non-feeding state), or roaming (exploring the environment). Worms generally spend most of the time dwelling, however, animals shifted to an environment lacking food start to roam after 30 min to explore for nutrients (Bargmann, 2006). Serotonin released from the NSM and HSN motor neurons induces dwelling, and PDF neuropeptide signaling from the AIY, RIM, and RIA interneurons induce prolonged roaming (Flavell et al., 2013). Food seeking behavior is also regulated by several other neurons including the ADF, ASE, ASI, AWC, and BAG (Gray et al., 2004; Wakabayashi et al., 2004; Juozaityte et al., 2017; Rhoades et al., 2019). ETS-5, which controls the BAG neurons gas-sensing ability, is also involved in regulating foraging behavior and fat metabolism. ETS-5 controls foraging behavior by regulating the expression of neuropeptides, including *flp-13* and *flp-19*, in the BAG and ASG neurons (Juozaityte et al., 2017). ETS-5 controls fat storage levels, which feeds back to control roaming and quiescence behaviors (Juozaityte et al., 2017). The AWC neurons also control foraging behavior by sensing volatile attractants from food. CEH-36, which is involved in AWC development, is also required for foraging. The lipid-TORC1 signaling pathway, including monomethyl branched-chain fatty acids from the intestine, induces CEH-36 expression during starvation, which promotes foraging (Figure 5A; Kniazeva et al., 2015).

#### Learning and Memory

Learning from and remembering experiences is critical for launching effective behavioral responses. *C. elegans* possess different learning processes, classified as: non-associative learning, associative learning, and imprinting. Learning from different environmental conditions, such as changes in temperature, gases, and odorants, establishes short- and long- term memories (Ardiel and Rankin, 2010). Various neurons mediate these processes, including the AWC, ASH, and AFD sensory neurons (Hawk et al., 2018; Eliezer et al., 2019) and the AIB, AIY, AVA, AVD, RIA, and RIM interneurons (Stetak et al., 2009; Jin et al., 2016).

Long-term memory formation is mediated by the CREB TF CRH-1, which has seven isoforms expressed across several tissues (Amano and Maruyama, 2011; Dahiya et al., 2019). The *crh-1e* isoform is expressed in RIM interneurons and is necessary for long-term memory formation of isoamyl alcohol exposure (Dahiya et al., 2019). CRH-1 is also involved in short-term memory formation against pathogens—where for a short time after conditioning to a pathogen, *C. elegans* tend to move toward that pathogen. After conditioning to *Staphylococcus aureus*, CRH-1 expression increases, and is required for chemotaxis toward *S. aureus* (Prithika et al., 2017). In another paradigm, animals that experience high or low salt concentrations during starvation learn to associate those salt concentrations with starvation, and therefore avoid them. This taste avoidance learning behavior is mediated by DAF-16, a FOXO TF that is the major target of insulin-like signaling in the ASER neuron. DAF-16 regulates neuropeptide production in ASER by controlling the expression of the neuropeptide processing enzyme EGL-21 (Figure 5B; Nagashima et al., 2019).

#### Pathogenic Avoidance Behavior

Distal tissues can act on the nervous system to control aversive learning behavior. The INS-11 insulin-like neuropeptide is an excellent example of this: where the presence of pathogenic bacteria increases *ins-11* expression in intestinal cells through the HLH-30 TF and p38 MAPK pathway (Lee and Mylonakis, 2017). INS-11 secreted from the intestine regulates *ins-6* (another insulin-like neuropeptide) and *tph-1* (involved in serotonin biosynthesis) in the ASI and ADF neurons, respectively, which can adjust aversive behavior to the bacteria (Lee and Mylonakis, 2017). *C. elegans* can prepare for a pathogenic bacterial attack, solely by detecting bacterial odor. This process is controlled by release of serotonin from serotonergic neurons, which causes HSF-1 localization in nuclear bodies where it controls the expression of chaperone genes such as *hsp-70* to prepare the animal against pathogens (Figure 5C; Ooi and Prahlad, 2017).

### Transcription Factors and Sex-Specific Behaviors

*C. elegans* has two sexes (self-fertilizing hermaphrodite and male) that have specific anatomy and physiology, to drive sex-specific behaviors. For example, hermaphrodites lay eggs, males perform mating behavior, and both sexes have specific olfactory behaviors (Lee and Portman, 2007). Sex-specific neuronal circuits drive these behaviors, including sex-specific neurons: 8 in hermaphrodites and 91 in males (Barrios et al., 2008). In addition, connections between neurons can be different between the sexes (Oren-Suissa et al., 2016; Cook et al., 2019; Molina-García et al., 2020), and a neuron may mediate sex-specific behavior depending on the sexual-context of the animal (Lee and Portman, 2007). Like other behaviors, developmental factors as well as environmental conditions, such as food availability, temperature and CO_2_ level can influence sex-specific behaviors (Gruninger et al., 2006; Fenk and de Bono, 2015; Gouvêa et al., 2015; Nett et al., 2019). Here we describe the sex determination pathway and TFs that regulate sex-specific behaviors by controlling neuron development and synaptogenesis.

#### Sex Status and Behavior

**FIGURE 6:**
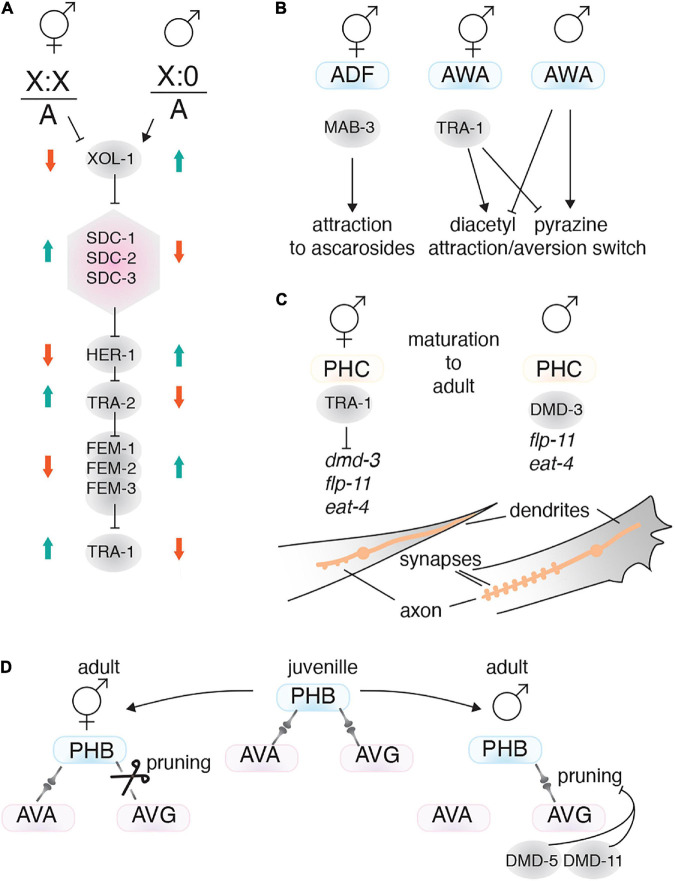
Transcription factors involved in sex-specific behavior. **(A)** The *C. elegans* sex determination pathway. The X chromosome/autosome ratio (X:0) controls the pathway by regulating XOL-1. In males, high XOL-1 activity supresses the pathway, resulting in lower TRA-1 expression. TRA-1 is the master regulator of the hermaphrodite phenotype. The dosage compensation complex is shown as a pink hexagon. **(B)** TFs (gray circles) that drive sex-specific chemotaxis. **(C)** The TFs TRA-1 and DMD-3 regulate sex-specific PHC neuron characteristics. The PHC tail neurons exhibit sex-specific axo-dendritic length and synapse number. **(D)** Sex-specific connectome formation is achieved through differential pruning. For instance, connections between PHB and AVG are pruned in hermaphrodites, but retained in males. Male-specific *dmd-5* and *dmd-11* expression suppresses synaptic pruning between the PHB and AVG neurons.

Sexual status impacts how the nervous system and behavioral programs develop. The sex determination pathway in *C. elegans* has three main players: X chromosome/autosome ratio (X:A), the master regulator XOL-1 and the Gli-type zinc finger TF TRA-1 (Wolff and Zarkower, 2008). In XX animals the X:A ratio is high, which supresses *xol-1*, leading to hermaphrodite sexual differentiation. Low XOL-1 levels triggers dosage compensation complex formation, which controls the level of X chromosome gene expression, represses expression of the secreted protein HER-1, and activates the sex-determining transmembrane factor TRA-2 (Figure 6A; Miller et al., 1988; Carmi and Meyer, 1999). In XO animals, the X:A ratio is low which leads to high XOL-1 levels. High XOL-1 triggers male sexual differentiation and inhibits assembly of the dosage compensation complex (Miller et al., 1988; Carmi and Meyer, 1999). Downstream of XOL-1, TRA-1 acts as the final step in the sex determination pathway. TRA-1 is active in XX animals and is required for the hermaphrodite phenotype, whereas TRA-1 repression in XO animals, by the FEM-1, FEM-2, and FEM-3 factors, is necessary for male phenotype formation (Figure 6A; Hodgkin and Brenner, 1977; Berkseth et al., 2013).

A useful feature of *C. elegans* sex determination is that it acts cell autonomously. Therefore, ectopically expressing FEM-3 to masculinize or TRA-2 to feminize a specific neuron can resolve how sexuality affects that neurons function. Mating behavior is a prominent example of sexual dimorphism. Mating success requires males to perceive pheromones (ascarosides) released from hermaphrodites, and to be attracted to them. In contrast, hermaphrodites are weakly repelled by pheromones (Ludewig and Schroeder, 2018). Males sense pheromones by CEM (male-specific) and ADF (sex-shared) neurons (Srinivasan et al., 2008; Fagan et al., 2018). Masculinizing or feminizing the ADF neurons can change mating behavior. Males with feminized ADF neurons are repelled by ascarosides, whereas hermaphrodites with masculinized ADF neurons are attracted to ascarosides (Fagan et al., 2018). The TF MAB-3 drives the masculine characteristics of ADF neurons in males and is required for attraction toward ascarosides. MAB-3 is expressed only in male ADFs and masculinizing the ADFs in hermaphrodites results in MAB-3 expression (Figure 6B; Fagan et al., 2018).

Another sexually dimorphic behavior is olfaction, where each sex shows different preferences to certain compounds. For instance, the sexuality of the AWA and AWC neurons can affect their preference to chemicals such as diacetyl and pyrazine (Lee and Portman, 2007). Masculinizing hermaphrodite animals, either by mutating *tra-1* or by driving *fem-3* using a pan-neuronal promoter, reverses olfactory preference. However, the genes and mechanisms that control olfaction preference in AWA and AWC downstream of the sex determination pathway remain to be determined (Figure 6B).

The PHC neurons are present in both sexes, but have sex-specific connectivity, morphology and physiology that are involved in male mating behavior. PHCs are highly connected with other neurons in males, whereas they are much less connected in hermaphrodites. The PHCs extend axons anteriorly and dendrites posteriorly. In immature animals, both sexes show similar morphology, but during maturation the PHC dendrites in males retract, whereas, in hermaphrodites they project to the tip of the tail (Serrano-Saiz et al., 2017). Also, during maturation the PHC axons in males grow anteriorly beyond the pre-anal ganglion, but in hermaphrodites they remain in an immature state. After maturation, male PHCs show a sex-specific expression pattern for some genes, including the neuropeptide *flp-11*, which is only expressed in L4 males (Serrano-Saiz et al., 2017). Male PHCs also express higher levels of the glutamate transporter *eat-4* compared to hermaphrodites (Serrano-Saiz et al., 2017). All of these sex-specific characteristics in male PHCs are controlled cell-autonomously through the sex determination pathway and the Doublesex DNA domain TF DMD-3. DMD-3, which is controlled by TRA-1, is expressed only in male PHCs and is necessary and sufficient to induce male-specific PHC features (Figure 6C; Serrano-Saiz et al., 2017).

#### Sex-Specific Neuron Circuits

*C. elegans* hermaphrodites and males possess sex-specific connectomes that control their behavior. The sex-specific connectome results from synaptic pruning during the later stages of development. Both sexes in the larval stages have a similar connectome, but during sexual maturation synapses not required for that sex are pruned (Oren-Suissa et al., 2016). Sex specific connectomes are regulated by the sex determination pathway, and cell-autonomous feminization or masculinization leads to the opposite synaptic pattern forming. Sex-shared neurons with sex-specific connectivity are related to sexually dimorphic behavior. For example, differences in the connections between the PHB and AVG sensory neurons control forward locomotion and chemo-repulsive behaviors in hermaphrodites, and mating behavior in males (Hilliard et al., 2002; Oren-Suissa et al., 2016). This process is mediated by the DMD-5 and DMD-11 TFs, which are only expressed in male AVG neurons, and are required for mating behavior in males (Oren-Suissa et al., 2016). DMD-5 and DMD-11 do not act in synapse formation but supress the pruning process in the neuron (Figure 6D; Oren-Suissa et al., 2016). In addition to sex-specific TFs, other TFs also regulate pruning, such as MBR-1/Mblk-1. MBR-1 expression is controlled by UNC-86 and is necessary for pruning synaptic connections between the AIM neurons. However, MBR-1’s role in behavior remains to be studied (Kage et al., 2005).

Experience during development can also influence synaptic pruning. For example, males starved during the L1 stage develop the hermaphrodite synaptic pattern between PHA and AVG and PHB and AVA, a process controlled by octopamine and serotonin signaling from the ADF neurons (Bayer and Hobert, 2018). These changes in synaptic pruning alter behaviors, such as enhanced chemosensory avoidance and mating (Bayer and Hobert, 2018).

## Perspective

We have described the TFs that play critical roles in defining the neuronal identities that control behavioral responses. Most of our knowledge about the molecular basis for behavior comes from studies in sensory neurons. Much of the molecular biology in other neurons, particularly interneurons which are the center of data processing and cognition, remains to be discovered. This bias toward sensory neurons is likely due to their relative ease of study. Interneurons, on the other hand, have large numbers of intersecting inputs and outputs from other neurons, making dissection of specific molecular pathways involved in a particular behavior a challenge. Further complexity arises when we consider differences between sexes or developmental stages. Our understanding of how neuronal architecture and neuron function differ between sexes has increased significantly in recent years (Jarrell et al., 2012; Oren-Suissa et al., 2016; Serrano-Saiz et al., 2017; Bhattacharya et al., 2019; Cook et al., 2019). Future studies using single-cell transcriptional profiling and CRISPR/Cas9 technology will expand on this knowledge to further understand what roles TFs play in establishing and maintaining these sex-specific differences. Beyond this, our understanding of how sex affects behavior is currently limited and needs to be expanded. Sexual dimorphism likely exists for most behaviors, as the underlying biological drive for behavior is different between the sexes—hermaphrodites prioritize food and egg laying, while males prioritize mating. Dissecting all the exquisite complexities of behavior, and the molecular mechanisms driving it, are entirely possible with the *C. elegans* model.

The majority of research so far has focused on how neuron function is established and maintained, such as neuron morphology, receptor expression, signaling pathways and synaptic patterning. However, organisms need to respond to changes they experience throughout their life. There is a need for continuous adaption, yet we know little of how TFs drive these adaptations. Very few examples of such mechanisms exist for a phenomenon that is fundamental for survival. Two examples we have are HIF-1 and DAF-16, which are involved in switching neuronal function developmentally. During hypoxia, HIF-1 activation in the ASG neurons induces serotonin synthesis, which enhances the animals response to hypoxia (Pocock and Hobert, 2010). This represents an adaptive sensory circuit that is not present under normoxic conditions. Dauer is an alternative developmental state that animals enter when food is absent. In the dauer state, the animal’s locomotion and chemosensory behaviors change, which relies on the plasticity of the electrical connectome. During dauer, DAF-16 regulates expression of the innexin protein INX-6 in the AIB neurons. INX-6, along with its partner homeobox TF CEH-7, are required for normal locomotion and chemotaxis behavior (Bhattacharya et al., 2019).

We understand that gene expression changes drive these adaptations, but how are the TFs themselves affected? More work is needed to understand how the levels, location, or activity status of TFs change in response to environmental signals. These changes would likely take place both developmentally and post developmentally, as environmental signals continue to change throughout an organism’s entire life. Alterations to TF activity would enable transient alterations to the gene expression program of a neuron. Reduced gene expression or altering protein stability can change the level of a TF in the cell. Altering a TF’s location is a common mechanism to control TF activity, DAF-16 in the insulin-like signaling pathway being a prime example of this: DAF-16 is maintained in the cytosol through phosphorylation when DAF-2 is activated and is nuclear localized when DAF-2 is inactive (Nagashima et al., 2019). The resulting gene expression changes can alter the levels of receptors that sense environmental stimuli and change the type or strength of neuropeptide or neurotransmitter signals produced by that neuron, thereby altering the animal’s behavioral response. Therefore, understanding how TFs act under different conditions, such as stress or high/low nutrients, and how neuronal function is altered under these conditions is a major challenge to address going forward. Again, developments in single-cell sequencing and tools such as the CRISPR/Cas9 and the auxin-induced degradation system (Zhang et al., 2015), will no-doubt greatly enhance our understanding of how TF dynamics control behavior and how this enables adaptation to an ever changing environment.

## Author Contributions

All authors listed have made a substantial, direct and intellectual contribution to the work, and approved it for publication.

## Conflict of Interest

The authors declare that the research was conducted in the absence of any commercial or financial relationships that could be construed as a potential conflict of interest.

## Publisher’s Note

All claims expressed in this article are solely those of the authors and do not necessarily represent those of their affiliated organizations, or those of the publisher, the editors and the reviewers. Any product that may be evaluated in this article, or claim that may be made by its manufacturer, is not guaranteed or endorsed by the publisher.
